# A case report of *NPHP1* deletion in Chinese twins with nephronophthisis

**DOI:** 10.1186/s12881-020-01025-x

**Published:** 2020-04-19

**Authors:** Feng Chen, Limeng Dai, Jun Zhang, Furong Li, Jinbo Cheng, Jinghong Zhao, Bo Zhang

**Affiliations:** 1 Department of Nephrology, the key Laboratory for the Prevention and Treatment of Chronic Kidney Disease of Chongqing, Kidney Center of PLA, Xinqiao Hospital, Army Medical University (Third Military Medical University), Chongqing, 400037 China; 2 Department of Medical Genetics, Army Medical University (Third Military Medical University), Chongqing, 400038 China

**Keywords:** Nephronophthisis, *NPHP1*, Exome sequencing, Autosomal recessive, Full gene deletion

## Abstract

**Background:**

Nephronophthisis (NPHP) is a rare autosomal recessive inherited disorder with high heterogeneity. The majority of NPHP patients progress to end-stage renal disease (ESRD) within the first three decades of life. As an inherited disorder with highly genetic heterogeneity and clinical presentations, NPHP still poses a challenging task for nephrologists without special training to make a well-judged decision on its precise diagnosis, let alone its mechanism and optimal therapy.

**Case presentation:**

A Chinese family with NPHP was recruited in current study. The clinical characteristics (including findings from renal biopsy) of NPHP patients were collected from medical records and the potential responsible genes were explored by the whole exome sequencing (WES). A homozygous deletion of *NPHP1* (1–20 exons) was found in both affected patients, which was further confirmed by quantitative PCR.

**Conclusions:**

Homozygous full gene deletion of the *NPHP1* gene was identified in a Chinese family with NPHP, which was the molecular pathogenic basis of this disorder. Furthermore, identification of the pathogenic genes for those affected patients can help to have a full knowledge on NPHP’s molecular mechanism and precise treatment.

## Background

Nephronophthisis (NPHP, OMIM 256100) is an autosomal recessive cystic kidney disease and is one of the most frequent genetic causes of renal failure in children [1]. The estimated incidence of NPHP in North American is about 1/50,000 to 1/100,000 [2]. A European study in 1998 reported an incidence of NPHP as 1 in 61,800 live births [3]. Based on the age of onset of renal failure, NPHP has been traditionally subdivided into infantile, juvenile and adolescent forms.

Typical clinical features of familial juvenile NPHP include polyuria, polydipsia and isosthenuria due to an impaired ability to concentrate urine and retain water. Ultrasonography is the most useful imaging technique for clinical diagnosis of NPHP. Typical ultrasound features include normal or reduced renal size, echogenicity with loss of corticomedullary differentiation and corticomedullary cysts. Histopathology study of renal biopsy reveals cortical microcysts, tubular atrophy, interstitial fibrosis and tubular basement membrane defects [4]. The disease always progresses to ESRD in childhood or early adolescence, with a need for renal replacement therapy. A small proportion of NPHP patients also display additional extrarenal abnormalities, such as liver fibrosis, situs inversus, or cardiac malformations. For example, retinitis pigmentosa and neurologic anomalies develop in Senior-Loken syndrome and Joubert syndrome respectively [5, 6].

The etiology of NPHP lies in the primary cilium. To date, NPHP and other cystic kidney diseases are referred to as ciliopathies based on the finding that all those proteins mutated in the cystic kidney diseases are expressed in the primary cilia, which are microtubule-based organelles that can be found on most human cell types. Although NPHP is genetically heterogeneous, the main cause of NPHP is the mutations in *NPHP1* gene, which encodes a protein with Src homology domain 3 [7]. NPHP1 protein can interact with Crk-associated substrate, whose function is associated with the control of cell division, cell-cell and cell-matrix adhesion signaling. It was reported that NPHP1 protein can function as part of a multifunctional complex such as primary cilia or centrosomes [8]. The largest proportion of NPHP patients (20–25%) is caused by homozygous full gene deletions of the *NPHP1* gene, as a result of recurrent complex rearrangements at this locus due to flanking low copy repeats [9].

For nephrologists, it is hard to diagnose the isolated NPHP patients. Recently, 26 patients with homozygous *NPHP1* deletions were identified among 5606 European patients with adult-onset ESRD, but only three (12%) of the 26 patients were classified as NPHP [10]. NPHP patients are easily misdiagnosed with other nephropathy or chronic kidney disease of unknown etiology, as no specific symptom could be found in NPHP patients. Establishing the diagnosis of NPHP based on clinical findings and renal ultrasound examination is far from enough. Therefore, it is important to perform genetic testing for suspected NPHP patients for its heterogeneity. Adhered to CARE guidelines/methodology, we reported a case of Chinese family with full gene deletion of *NPHP*1, which was detected by WES in this study.

## Case presentation

### Patients and clinical evaluation

A 16-year-old Chinese Han female was admitted to our Department of Nephrology for abnormal renal function. About 10 days ago, she suffered from abdominal pain and was treated as gastritis at other hospital. Abnormal renal function was detected at that hospitalization. She had no personal history of severe infectious diseases such as hepatitis and tuberculosis or family history of kidney disease, and she also denies smoking or drinking.

She had anemic appearance without edema, while the heart rate and blood pressure were normal. No obvious abnormality including growth retardation was detected during the physical examination, and no specific symptoms of NPHP such as polyuria and polydipsia were recognized. Blood routine test showed decreased blood cells and haemoglobin (Table 1). Serum chemistry showed elevated levels of serum creatinine, uric acid, cystatin-C and parathyroid hormone. The estimated glomerular filtration rate (eGFR) was 25.32 ml/min/1.73m^2^ without proteinuria. In order to confirm previous diagnosis, abdominal ultrasound examination was performed. Both kidneys were of normal size, but had hyperechogenicity. Renal cysts were detected on the right side. No abnormality was detected in the liver, pancreas and spleen by ultrasound examination.

**Table 1:** Abnormal laboratory data at presentation

Parameters	Proband	Affected Sib	Reference range
Blood routine tests			
WBC,10^9^/L	**2.98↓**	**3.27↓**	3.5–9.5
HGB,g/L	**80↓**	**83↓**	115–150
Platelet count,10^9^/L	**100↓**	**107↓**	125–350
RBC,10^12^/L	**2.81↓**	**2.92↓**	3.8–5.1
Urine routine tests			
Specific gravity	**1.010↓**	**1.010↓**	1.015–1.030
Serum chemistry			
Serum creatinine,μmol/L	**243↑**	**234.4↑**	45–105
Serum uric acid,μmol/L	**463.1↑**	**443.1↑**	140–420
Cystatin-C,mg/L	**2.66↑**	**2.98↑**	0–1.16
PTH,pg/ml	**724.1↑**	**469.4↑**	12–65

Blood values in result column indicate the abnormaldetected valued out of the reference range

**Fig. 1:**
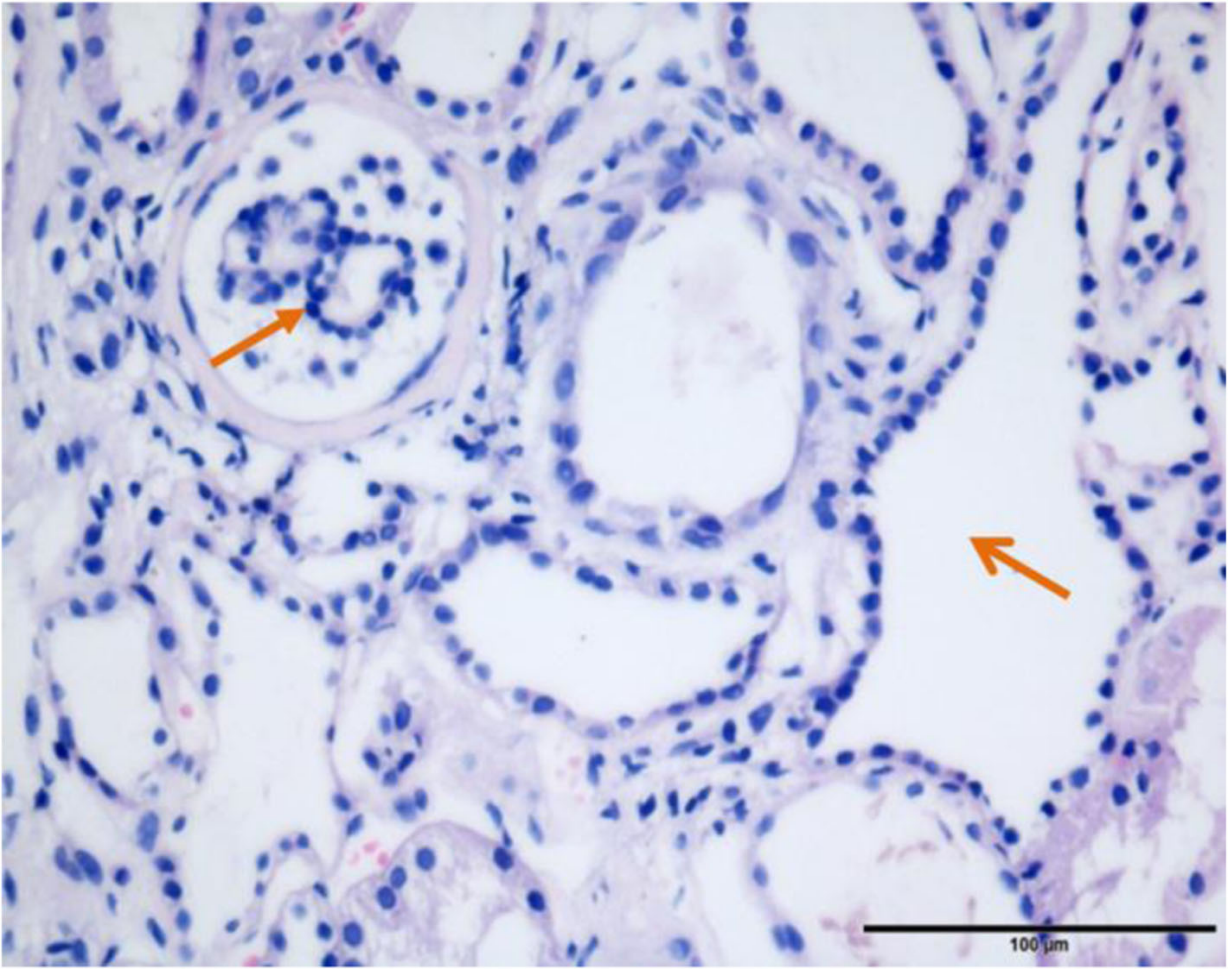
Histopathologic lesions. PAS staining showed the shrunk glomeruli and renal tubular dilation

To further understand its renal pathology, histopathology study of renal biopsy was performed. Totally, 21 glomeruli were observed, with 8 glomeruli being ischemic sclerosis. Partial glomeruli were shrunk, with the matrix showing slight hyperplasia. Focal tubular atrophy was observed, while dilatation and hypertrophy of partial renal tubules were also detected (Fig.1). The immunological staining was totally negative. All these changes indicated renal dysplasia.

Considering the possibility of genetic factor involved, the twin sister of the proband was recommended for serum chemistry test. Unfortunately, the elevated levels of serum creatinine, uric acid, cystatin-C and parathyroid hormone were detected (Table 1). Also, decreased blood cells and haemoglobin were detected by blood routine test. The results of other tests were similar to those of the proband.

### Genetic analysis

**Fig. 2:**
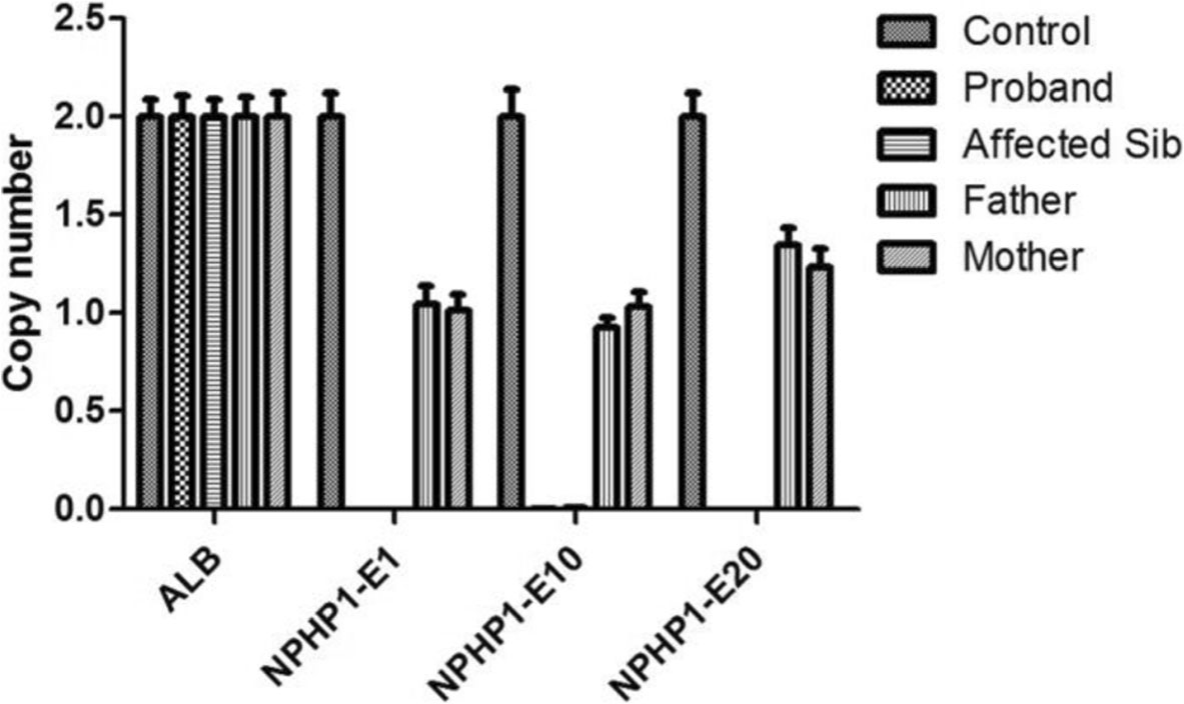
Copy number of *NPHP1* gene was determined by Quantitative PCR

As both twin had similar clinical presentations, genetic testing was the best way to find its molecular basis. Thus WES was performed and homozygous full gene deletion of the *NPHP1* gene was found in both affected twins. By comparing the relative reads depth of sequencing, a deletion spanned chr2:110881367–110,962,545 (hg19) was found. This deletion contains exon 1 to 20 of NPHP1 gene, and is pathogenic. To further confirm the full gene deletion, realtime PCR was performed to detect the copy number of this region, normalized by that of *albumin (ALB)* gene in human genome. As shown in Fig.2, only one copy of *NPHP1* gene was detected in the samples of their parents (Supplementary material).

## Discussion and conclusions

NPHP is a rare genetic disorder with highly variable clinical presentations and genetic heterogeneity. The majority of juvenile NPHP patients will develop ESRD between the ages 11 to 13 years [11]. Diagnosis of NPHP in adult patients is very uncommon. Bollée G et al. reported that four cases of adult NPHP patients were diagnosed through the identification of NPHP1 gene deletion [12]. Due to an impaired ability to concentrate urine and retain water, most patients present typical nephronophthisis symptoms such as polyuria, polydipsia and nocturnal enuresis, as well as progressive renal failure. Typical findings of ultrasound examination include increased echogenicity of the kidneys and reduced corticomedullary differentiation, renal cyst formation on the corticomedullary border. Our case showed atypical symptoms via both clinical findings and ultrasound examination. However, based on these clues, the nephrologists cannot establish a clear diagnosis at first sight. As conditions associated with renal cysts can mimic the NPHP phenotype, it is important to make a differential diagnosis of NPHP with other renal cystic diseases such as polycystic kidney disease (PKD), renal cysts and diabetes (RCAD). The almost same clinical manifestation of her twin sister strongly suggested that there may be some forms of inherited renal cystic disease that remain undiscovered.

Although NPHP is of high heterogeneity, the main genetic cause of NPHP is the mutations in *NPHP1* gene, and homozygous deletions in the *NPHP1* gene are the major contributor. Therefore, gene testing of *NPHP1* based on PCR is most important for patients with suspected NPHP. By using this method, Hussain et al. found the homozygous deletion of the *NPHP1* gene in 6 unrelated Pakistani families [13]. In a cohort of 35 Japanese patients, large deletions of NPHP1 gene frequently were determined by PCR with a set of primers [14]. Other techniques for detecting large deletions of NPHP1 gene include fluorescence in situ hybridization [15] and multiplex ligation-dependent probe amplification (MLPA) [11]. With the tremendous progresses of next generation sequencing (NGS), WES is more efficient than other techniques. This method has been extensively applied for gene testing of inherited diseases including NPHP. Recently, Tang X et al. analyzed gene mutation and clinical manifestations of NPHP in Chinese population by using WES, and found the causative genetic variations in these patients [16]. Also, targeted exome sequencing (TES) was used to detect the causative mutation of NPHP patients. For example, TES based on 63 ciliopathy genes was performed in the probands of the two Chinese pedigrees of NPHP [17]. In a case report, the causative mutation of NPHP patient was detected by NGS based on a panel of 21 genes [18]. All these studies demonstrated that NGS is a powerful technique to identify gene mutations in patients with NPHP.

To date, there are more than 300 variants of *NPHP1* gene deposited in the ClinVar database, most of which are associated with copy number variants such as duplication and deletion of large fragment. NPHP1 gene is flanked by two large inverted repeats of approximately 330 kb in size, which leads to an unequal recombination [19]. Other types of mutations, such as misssense, nonsense and splice site mutation, are also included in the database. Due to the relative low morbidity, the number of NPHP patients in clinical study is rather small. Thus the genotype-phenotype correlation of NPHP is not clear. A study of Japanese NPHP patients showed that an NPHP gene (including NPHP1, NPHP3, and NPHP4) mutation was identified in more than 50% of the 35 NPHP patients [14]. In a Dutch cohort of 40 patients, NPHP1 was the most frequently mutated gene in the cohort, while other genes included NPHP4, WDR35, BBS1, AHI1, BBS10, IQCB1 and OFD1 [20]. In another Germany study, monogenetic defects of NPHP genes were identified in 97 of 152 patients, and homozygous NPHP1 deletion was the most frequent genetic defect [21]. Although patients with different gene mutations showed somewhat different symptoms, the conclusive gene-phenotype associations need further investigation. All these studies suggest that NPHP is genetically and phenotypically heterogeneous. Therefore, it is necessary to identify the pathogenic variants by WES. Our case is caused by homozygous *NPHP1* deletion, which resembles those reported in Western populations.

The onset age of NPHP ranges from infantile to adult. It has been hypothesized that this non-pediatric onset of ESRD, especially after 30 years old, is due to the influence of yet unknown modifier genes. The onset age of ESRD was reported to be 27 to 43 year-old in a Turkish family, in which modifier genes might be involved [22]. For NPHP patients, no modifier gene has been identified. However, a recent case report showed that PKD1 mutation might epistatically ameliorate NPHP progression in patients with NPHP1 deletion [23]. In our case, no such NPHP-modifier variant was observed.

In summary, current study indicates that the clinical diagnosis of atypical NPHP patient is difficult. Our case was verified to be homozygous full gene deletion in *NPHP1* gene by WES. The mutation in this case helps to understand the relationship between genotype and phenotype of NPHP. In addition, WES is suitable for genetic diagnosis of inherited diseases like NPHP.

## Supplementary information

**Additional file 1.**

## Data Availability

All data generated or analyzed during this study are available from the corresponding author upon reasonable request.

## Abbreviations

NPHP: Nephronophthisis
ESRD: End-stage renal disease
WES: Whole exome sequencing
eGFR: Glomerular filtration rate
ALB: Albumin
TES: Targeted exome sequencing
