# Supplementary material for: A case report of NPHP1 deletion in Chinese twins with nephronophthisis
Source: BMC Med Genet. 2020 Apr 19;21:84. doi: 10.1186/s12881-020-01025-x (PMC7168837; doi:10.1186/s12881-020-01025-x)

**Supplementary material**

**Copy number detection**

Blood samples of the proband and her family were collected into graded negative pressure vacuum EDTA anticoagulant tubes. Genomic DNA was isolated from the peripheral blood cells with a QIAamp DNA Blood MiniKit (Qiagen, Germany), according to the manufacturer’s instructions. DNA of health control was used as positive control. *Albumin* (*ALB*) gene, whose putative copy number was 2 in human genome, was taken as reference gene of *NPHP1*. Then realtime PCR amplification was done with a 10-μL final reaction mixture consisting of 50ng genomic DNA, 0.1 μM of the each sense and antisense primers (below), and 1× PCR mixture with SYBR Green I (BioRad, China), using the CFX96 Real-Time System (Bio-Rad). The PCR conditions were initial denaturation at 95 °C for 10 min, 45 cycles of denaturation at 95 °C for 20 s, annealing at 60 °C for 20 s, and elongation at 72 °C for 40 s. The relative copy number of *NPHP1* gene was normalized by that of *ALB* gene.

PCR primers are list below.

1QF: ACCGCAAGAGAACATTTGACCCT

1QR: GTCGTCTCGCCAGCATCTCC

10QF: GGGAATCAATTTCGAGCAAATTACTTC

10QR: CTGATAGTAACTATACTTACAGTGCCTT

20QF: GTTTACCATGACTGCGTGCTCCC

20QR: GTGATAACTTTCCACCGTGCAGTCT

Raw data

| **Reaction 1-Proband** | | |  |  | |  | |
| --- | --- | --- | --- | --- | --- | --- | --- |
| Sample Name | Target Name | Cт | | Cт Mean | Cт SD | |  |
| Proband | ALB-Q | 24.60289383 | | 24.64263916 | 0.056358654 | |  |
| Proband | ALB-Q | 24.6178894 | | 24.64263916 | 0.056358654 | |  |
| Proband | ALB-Q | 24.70713997 | | 24.64263916 | 0.056358654 | |  |
| Healthy Control | ALB-Q | 24.04169464 | | 24.01793098 | 0.025743397 | |  |
| Healthy Control | ALB-Q | 23.99058342 | | 24.01793098 | 0.025743397 | |  |
| Healthy Control | ALB-Q | 24.02151489 | | 24.01793098 | 0.025743397 | |  |
| Proband | NPHP1-1Q | 35.92437553 | | 35.74179586 | 0.139988023 | |  |
| Proband | NPHP1-1Q | 35.71678925 | | 35.74179586 | 0.139988023 | |  |
| Proband | NPHP1-1Q | 35.58422279 | | 35.74179586 | 0.139988023 | |  |
| Healthy Control | NPHP1-1Q | 22.45413208 | | 22.4806366 | 0.045069572 | |  |
| Healthy Control | NPHP1-1Q | 22.45510483 | | 22.4806366 | 0.045069572 | |  |
| Healthy Control | NPHP1-1Q | 22.5326767 | | 22.4806366 | 0.045069572 | |  |
| Proband | NPHP1-10Q | 33.99975662 | | 33.96756388 | 0.028496025 | |  |
| Proband | NPHP1-10Q | 33.93047333 | | 33.96756388 | 0.028496025 | |  |
| Proband | NPHP1-10Q | 33.9724617 | | 33.96756388 | 0.028496025 | |  |
| Healthy Control | NPHP1-10Q | 23.74986839 | | 23.83551025 | 0.118972704 | |  |
| Healthy Control | NPHP1-10Q | 23.78530502 | | 23.83551025 | 0.118972704 | |  |
| Healthy Control | NPHP1-10Q | 23.97135544 | | 23.83551025 | 0.118972704 | |  |
| Proband | NPHP1-20Q | 34.85512924 | | 34.72952588 | 0.088817005 | |  |
| Proband | NPHP1-20Q | 34.66599083 | | 34.72952588 | 0.088817005 | |  |
| Proband | NPHP1-20Q | 34.66745758 | | 34.72952588 | 0.088817005 | |  |
| Healthy Control | NPHP1-20Q | 22.57273674 | | 22.69268227 | 0.198560998 | |  |
| Healthy Control | NPHP1-20Q | 22.58343506 | | 22.69268227 | 0.198560998 | |  |
| Healthy Control | NPHP1-20Q | 22.92187881 | | 22.69268227 | 0.198560998 | |  |

| **Reaction 2-Father** | | |  | |  | |  | |
| --- | --- | --- | --- | --- | --- | --- | --- | --- |
| Sample Name | Target Name | Cт | | Cт Mean | | Cт SD | |  |
| Father | ALB-Q | 24.27997398 | | 24.30827522 | | 0.068320632 | |  |
| Father | ALB-Q | 24.25865173 | | 24.30827522 | | 0.068320632 | |  |
| Father | ALB-Q | 24.38619804 | | 24.30827522 | | 0.068320632 | |  |
| Healthy Control | ALB-Q | 23.7142334 | | 23.69408417 | | 0.031468537 | |  |
| Healthy Control | ALB-Q | 23.65782166 | | 23.69408417 | | 0.031468537 | |  |
| Healthy Control | ALB-Q | 23.71019554 | | 23.69408417 | | 0.031468537 | |  |
| Father | NPHP1-1Q | 24.91584969 | | 24.87973595 | | 0.034034587 | |  |
| Father | NPHP1-1Q | 24.84825516 | | 24.87973595 | | 0.034034587 | |  |
| Father | NPHP1-1Q | 24.875103 | | 24.87973595 | | 0.034034587 | |  |
| Healthy Control | NPHP1-1Q | 23.30104256 | | 23.33297539 | | 0.02769381 | |  |
| Healthy Control | NPHP1-1Q | 23.35040474 | | 23.33297539 | | 0.02769381 | |  |
| Healthy Control | NPHP1-1Q | 23.34748077 | | 23.33297539 | | 0.02769381 | |  |
| Father | NPHP1-10Q | 25.87737083 | | 25.90440941 | | 0.043158758 | |  |
| Father | NPHP1-10Q | 25.88167191 | | 25.90440941 | | 0.043158758 | |  |
| Father | NPHP1-10Q | 25.95418167 | | 25.90440941 | | 0.043158758 | |  |
| Healthy Control | NPHP1-10Q | 24.21875191 | | 24.1814518 | | 0.036238477 | |  |
| Healthy Control | NPHP1-10Q | 24.14637756 | | 24.1814518 | | 0.036238477 | |  |
| Healthy Control | NPHP1-10Q | 24.17922592 | | 24.1814518 | | 0.036238477 | |  |
| Father | NPHP1-20Q | 24.90585136 | | 24.87044716 | | 0.030758426 | |  |
| Father | NPHP1-20Q | 24.85029221 | | 24.87044716 | | 0.030758426 | |  |
| Father | NPHP1-20Q | 24.85519981 | | 24.87044716 | | 0.030758426 | |  |
| Healthy Control | NPHP1-20Q | 23.71529198 | | 23.68429375 | | 0.03360546 | |  |
| Healthy Control | NPHP1-20Q | 23.6890049 | | 23.68429375 | | 0.03360546 | |  |
| Healthy Control | NPHP1-20Q | 23.64857864 | | 23.68429375 | | 0.03360546 | |  |

| **Reaction 3-Mother** | | |  | |  | |  | |
| --- | --- | --- | --- | --- | --- | --- | --- | --- |
| Sample Name | Target Name | Cт | | Cт Mean | | Cт SD | |  |
| Mother | ALB-Q | 23.39303207 | | 23.39689064 | | 0.016611885 | |  |
| Mother | ALB-Q | 23.38254547 | | 23.39689064 | | 0.016611885 | |  |
| Mother | ALB-Q | 23.41509056 | | 23.39689064 | | 0.016611885 | |  |
| Healthy Control | ALB-Q | 24.05611038 | | 24.03996468 | | 0.021078648 | |  |
| Healthy Control | ALB-Q | 24.04766655 | | 24.03996468 | | 0.021078648 | |  |
| Healthy Control | ALB-Q | 24.016119 | | 24.03996468 | | 0.021078648 | |  |
| Mother | NPHP1-1Q | 23.88141251 | | 23.90335274 | | 0.033945903 | |  |
| Mother | NPHP1-1Q | 23.94245338 | | 23.90335274 | | 0.033945903 | |  |
| Mother | NPHP1-1Q | 23.88619423 | | 23.90335274 | | 0.033945903 | |  |
| Healthy Control | NPHP1-1Q | 23.61193466 | | 23.56378174 | | 0.089407258 | |  |
| Healthy Control | NPHP1-1Q | 23.46061707 | | 23.56378174 | | 0.089407258 | |  |
| Healthy Control | NPHP1-1Q | 23.61878777 | | 23.56378174 | | 0.089407258 | |  |
| Mother | NPHP1-10Q | 25.43206215 | | 25.48993874 | | 0.054299291 | |  |
| Mother | NPHP1-10Q | 25.49799538 | | 25.48993874 | | 0.054299291 | |  |
| Mother | NPHP1-10Q | 25.53976059 | | 25.48993874 | | 0.054299291 | |  |
| Healthy Control | NPHP1-10Q | 25.15567398 | | 25.17933655 | | 0.025135441 | |  |
| Healthy Control | NPHP1-10Q | 25.20572281 | | 25.17933655 | | 0.025135441 | |  |
| Healthy Control | NPHP1-10Q | 25.17661095 | | 25.17933655 | | 0.025135441 | |  |
| Mother | NPHP1-20Q | 24.23616219 | | 24.19202995 | | 0.091292836 | |  |
| Mother | NPHP1-20Q | 24.25287247 | | 24.19202995 | | 0.091292836 | |  |
| Mother | NPHP1-20Q | 24.08705711 | | 24.19202995 | | 0.091292836 | |  |
| Healthy Control | NPHP1-20Q | 24.16552544 | | 24.13435936 | | 0.103861645 | |  |
| Healthy Control | NPHP1-20Q | 24.01848221 | | 24.13435936 | | 0.103861645 | |  |
| Healthy Control | NPHP1-20Q | 24.21906853 | | 24.13435936 | | 0.103861645 | |  |

| **Reaction 4-Sister** | | |  | |  | |  | |
| --- | --- | --- | --- | --- | --- | --- | --- | --- |
| Sample Name | Target Name | Cт | | Cт Mean | | Cт SD | |  |
| Sister | ALB-Q | 25.06304169 | | 25.01683998 | | 0.063530788 | |  |
| Sister | ALB-Q | 24.94439125 | | 25.01683998 | | 0.063530788 | |  |
| Sister | ALB-Q | 25.04308701 | | 25.01683998 | | 0.063530788 | |  |
| Healthy Control | ALB-Q | 24.64227486 | | 24.61648369 | | 0.025404643 | |  |
| Healthy Control | ALB-Q | 24.61569214 | | 24.61648369 | | 0.025404643 | |  |
| Healthy Control | ALB-Q | 24.59148407 | | 24.61648369 | | 0.025404643 | |  |
| Sister | NPHP1-1Q | 38.20980644 | | 38.18734639 | | 0.049947427 | |  |
| Sister | NPHP1-1Q | 38.11811829 | | 38.18734639 | | 0.049947427 | |  |
| Sister | NPHP1-1Q | 38.23411446 | | 38.18734639 | | 0.049947427 | |  |
| Healthy Control | NPHP1-1Q | 23.66203117 | | 23.58509636 | | 0.07046888 | |  |
| Healthy Control | NPHP1-1Q | 23.52368164 | | 23.58509636 | | 0.07046888 | |  |
| Healthy Control | NPHP1-1Q | 23.56957245 | | 23.58509636 | | 0.07046888 | |  |
| Sister | NPHP1-10Q | 32.62720871 | | 32.69709778 | | 0.06170075 | |  |
| Sister | NPHP1-10Q | 32.72004318 | | 32.69709778 | | 0.06170075 | |  |
| Sister | NPHP1-10Q | 32.74403381 | | 32.69709778 | | 0.06170075 | |  |
| Healthy Control | NPHP1-10Q | 24.62729454 | | 24.59743309 | | 0.025871821 | |  |
| Healthy Control | NPHP1-10Q | 24.58180237 | | 24.59743309 | | 0.025871821 | |  |
| Healthy Control | NPHP1-10Q | 24.58319664 | | 24.59743309 | | 0.025871821 | |  |
| Sister | NPHP1-20Q | 36.93940735 | | 36.70790609 | | 0.183183739 | |  |
| Sister | NPHP1-20Q | 36.69285202 | | 36.70790609 | | 0.183183739 | |  |
| Sister | NPHP1-20Q | 36.49145889 | | 36.70790609 | | 0.183183739 | |  |
| Healthy Control | NPHP1-20Q | 23.98997879 | | 23.96663475 | | 0.045737237 | |  |
| Healthy Control | NPHP1-20Q | 23.91393471 | | 23.96663475 | | 0.045737237 | |  |
| Healthy Control | NPHP1-20Q | 23.99598694 | | 23.96663475 | | 0.045737237 | |  |

Visualization of data


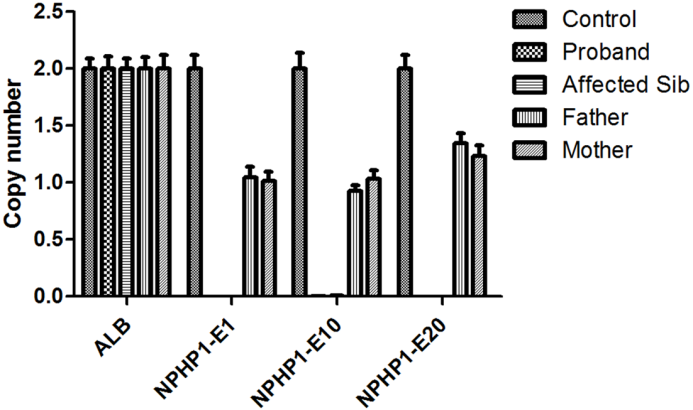

Supplement: Supplementary file 1 — Additional file 1. [file 12881_2020_1025_MOESM1_ESM.docx]
